# Psychological Impact of the Civil War and COVID-19 on Libyan Medical Students: A Cross-Sectional Study

**DOI:** 10.3389/fpsyg.2020.570435

**Published:** 2020-10-26

**Authors:** Muhammed Elhadi, Anis Buzreg, Ahmad Bouhuwaish, Ala Khaled, Abdulmueti Alhadi, Ahmed Msherghi, Ahmed Alsoufi, Hind Alameen, Marwa Biala, Alsafa Elgherwi, Fatimah Elkhafeefi, Amna Elmabrouk, Abdulmuez Abdulmalik, Sarah Alhaddad, Moutaz Elgzairi, Ahmed Khaled

**Affiliations:** ^1^Faculty of Medicine, University of Tripoli, Tripoli, Libya; ^2^Faculty of Medicine, Tobruk University, Tobruk, Libya; ^3^Faculty of Medicine, University of Zawia, Zawiya, Libya; ^4^Faculty of Medicine, University of Benghazi, Benghazi, Libya

**Keywords:** depression, PHQ-9, anxiety, GAD-7, medical student, COVID-19, SARS-CoV-2, civil war

## Abstract

**Objective:**

We aim to determine the psychological status of medical students during the COVID-19 outbreak and civil war in Libya.

**Methods:**

A cross-sectional study was conducted among medical students from 15 medical schools between April 20 and May 1, 2020. The demographic characteristics, generalized anxiety disorder 7-item (GAD-7) scale, and patient health questionnaire (PHQ-9) results were collected.

**Results:**

Of the 3,500 students, 2,430 completed the survey. The mean (± SD) score of anxiety symptoms determined by the GAD-7 was 7.2 (5.1). A total of 268 (11%) students had a GAD-7 score of ≥15, which is indicative of moderate to severe anxiety. A total of 1,568 (64.5%) students showed different degrees of anxiety: mild, 910 (37.5%); moderate, 390 (16%); and severe, 268 (11%). Anxiety was significantly associated with living status and internal displacement (*P* < 0.05). The mean (+ SD) score of depressive symptoms determined by the PHQ-9 was 9.7 (6.3). A total of 525 (21.6%) students had a PHQ-9 score of ≥15, which is indicative of moderate to severe depression. A total of 1,896 (88%) students were diagnosed with mild (PHQ ≥ 5) depression. Suicidal ideation was present in 552 patients (22.7%). Depression was only statistically associated with the year of study (*P* = 0.009).

**Conclusion:**

These data highlight that medical students in Libya are at risk for depression, especially under the current stressful environment of the civil war and the COVID-19 outbreak.

## Introduction

Severe acute respiratory syndrome coronavirus 2 (SARS-CoV-2) was identified in the city of Wuhan, Hubei Province, China in December 2019 (Chan et al., 2020; Zhou F. et al., 2020; Zhou P. et al., 2020). It causes severe viral pneumonia that has been designated COVID-19. Since December 2019, it has spread rapidly, and the World Health Organization (WHO) declared it a worldwide pandemic in February 2020 (Mahase, 2020; World Health Organization, 2020). On August 26, 2020, the WHO announced that 23.9 million people were infected and more than 820,000 had died (Dong et al., 2020; Huang et al., 2020).

Because of COVID-19, medical schools have suspended their function and closed for this period, and some countries have applied social distancing measures and curfews. These preventive measures may have increased stress and psychological pressure on medical students as they are unable to complete their studies and are at high risk of being infected with SARS-CoV-2 (Lai et al., 2020; Li et al., 2020; Reger et al., 2020; Wang et al., 2020). Addressing potential stressors during this time is important; possible disease exposure, economic privatization, and decreased social support may carry an increased risk for suicide and decreased mental and physical performance (Chandratre, 2020; Ullah and Amin, 2020).

Medical students are at higher risk of depression and anxiety for several reasons, including mentally and emotionally demanding medical school programs (Wolf, 1994), financial pressure, high workload, and sleep deprivation. In addition, exposure to sick and dead people can have a negative impact on their mental health (Wolf et al., 1988; Guthrie et al., 1998; Williams et al., 2005; Sreeramareddy et al., 2007; Boland et al., 2016). These demanding conditions put students in high stress positions, with anxiety being reported in one-third of medical students worldwide, especially those who reside in the Middle East and Asia (Quek et al., 2019). Other studies have reported a prevalence of up to 65.5% for anxiety and 66.5% for depression outside of North America (Hope and Henderson, 2014).

Medical schools in Libya use the 7-year program, comprising a preparatory year of biomedical science, followed by a 5-year program of 3 years of basic science and 2 years of clinical science, and 1 year of mandatory internship training.

Since 2011, Libya has suffered from several civil wars and conflicts with militias, causing increased insecurity and financial crises, as well as the kidnapping, rape, and killing of innocent people (Zeiton, 2011). Furthermore, the lack of funds for mental health services puts Libyan people at higher risk of mental disorders (Charlson et al., 2012; Abuazza, 2013; Rhouma et al., 2016), especially in large cities. Conflicts in the country have caused more than 217,000 people to be internally displaced, according to the United Nations Refugee Agency (UNHCR) report (Miller and Rasmussen, 2010; Reed et al., 2012; Newnham et al., 2015; UNHCR, 2020). In addition, they have affected the medical education system in Libya, several medical schools were temporarily closed for several periods during the war, which resulted in delays in the graduation and medical education of thousands of medical students. These stressors can have substantial negative effects on the psychological statuses of students.

Since the emergence of the first case of COVID-19 in Libya on March 24, 2020 (Elhadi et al., 2020a), the number of COVID-19 cases has substantially increased in many cities, with up to 11,834 confirmed cases and more than 210 deaths by August 26, 2020. The unpreparedness of the Libyan healthcare system toward the COVID-19 pandemic, which can be explained by the large number of cases and shortage of medical supplies, as well as the ongoing conflict, have placed an increased burden on Libyan medical students and the general population; the civil war has caused a financial crisis and reduced the ability of the healthcare system to provide adequate training opportunities for newly graduated medical students (Elhadi and Msherghi, 2020; Elhadi et al., 2020b). This, coupled with the closure of medical schools during the COVID-19 pandemic, along with extended electrical blackout issues, may have resulted in substantial frustrations and increases in anxiety and depression among medical students, especially for those in high-conflict areas or who were internally displaced from their homes.

Today’s medical students are tomorrow’s doctors; therefore, their mental wellbeing is crucial. However, few studies have addressed the impact of the COVID-19 pandemic on the mental health of medical students (Cao et al., 2020; Soled et al., 2020; Ullah and Amin, 2020). There is an urgent need to perform high-quality research and collect data on the effects of the COVID-19 outbreak on mental health (Holmes et al., 2020). Therefore, we aimed to determine the psychological status of medical students during the COVID-19 outbreak and civil war in Libya and determine factors associated with depression and anxiety among Libyan medical students.

## Materials and Methods

### Study Design

This is a cross-sectional study of medical students from 15 medical schools and colleges in Libya, located in the main cities. Data were collected from April 20, 2020 to May 1, 2020. Data were collected in paper and electronic forms using anonymous surveys that were sent to medical students by email and through the social media groups of medical students. The questionnaires were anonymous to ensure the reliability and correctness of the data, and were sent to more than 3,500 medical students. To avoid observer and selection bias, the survey was blinded: for the paper survey, the students completed the survey anonymously without identifiable data and left the completed paper survey at a designated collection point in each medical school to avoid any potential bias. For the online version, the students were asked to fill out the survey anonymously without identifiable data.

### Study Participants and Settings

Active medical students who were currently enrolled in medical schools were included, while those who were not currently enrolled in medical school were excluded. Those with a previous history of mental illness or missing data were excluded from the study. Students of any age and gender were included. The survey was conducted in the following cities: Tripoli, Al-Zawia, Misrata, Sebha, Gharyan, Albayda, Benghazi, Al-Khums, Tarhuna, Alzintan, Tobruk, and Sabratha.

### Study Tools

The questionnaire was divided into three parts. The first part contained demographic characteristics, including age, gender, year of study, availability of steady financial sources, living status (living alone or with family), presence of friends or family infected with COVID-19 infection that they know, and occurrence of internal displacement due to the civil war.

The second part was comprised of the generalized anxiety disorder 7-item (GAD-7) scale. This tool includes questions regarding seven anxiety symptoms and their frequencies within the last 2 weeks. It has a specificity of 82% and a sensitivity of 89% (Spitzer et al., 2006; Toussaint et al., 2020), and has been validated in several previous studies and has excellent internal consistency (Cronbach’s α = 0.83–0.93). In each of the questions students were asked to rate symptoms using a 4-item Likert scale of 0 (not at all), 1 (several days), 2 (more than half of the days), and 3 (nearly every day), with a total score of 0–21 (Ruiz et al., 2011; Johnson et al., 2019). A score of 0–4 was regarded as normal, 5–9 was regarded as mild, 10–14 was regarded as moderate, and 15–21 was regarded as severe (Kroenke et al., 2007). A GAD-7 score of ≥15 was regarded as the cutoff score to detect anxiety symptoms (Spitzer et al., 2006).

The third part consisted of the patient health questionnaire (PHQ-9), which is a validated 9-item questionnaire to assess depression severity in individuals. It has a specificity of 85% and a sensitivity of 88% (Levis et al., 2019). Each of the 9-item questions rates depression using a 4-item Likert scale of 0 (not at all), 1 (several days), 2 (more than half the days), and 3 (nearly every day), with a total score of 0–27 (Kroenke et al., 2001). A score of 0–4 was regarded as minimal, 5–9 was regarded as mild, 10–14 was regarded as moderate, 15–19 was regarded as moderately severe, and 20–27 was regarded as severe (Urtasun et al., 2019). A PHQ-9 score of ≥15 was regarded as the cutoff score to detect depressive symptoms (Kroenke et al., 2001; Levis et al., 2019).

### Statistical Analysis

The independent-samples *t*-test was used to determine if there was a significant difference between the means of the two groups. The chi-square test for association was used to determine the association between categorical groups. Spearman’s rank-order correlation was used to determine the association between continuous/ordinal variables. A *P*-value of less than 0.05 was considered statistically significant. Statistical analyses were performed using IBM SPSS Statistics for Windows (Version 25.0).

### Ethical Standards

The authors assert that all procedures contributing to this work comply with the ethical standards of the relevant national and institutional committee on human experimentation with the Helsinki Declaration of 1975, as revised in 2008. Ethical approval for this study was obtained from the Bioethics Committee at the Biotechnology Research Center in Libya. All participants provided consent before participating in the study.

## Results

### Demographic Characteristics

A total of 2,430 out of 3,500 students completed the survey. Of these, 1,327 (54.6%) were from the University of Tripoli. The mean age of the study participants was 23.30 ± 2.61 years, and 734 (30.2%) students were in the 5th year of their medical course. The vast majority (1,919 out of 2,430 [78.97%]) were female, and 511 (21.03%) were male. Table 1 provides the demographic characteristics of the study participants.

**TABLE 1:** Characteristics of students in the study.

Variable	Total *n* = 2,430	Female *n* = 1,919	Male *n* = 511	*P*-value
Age (mean ± SD)	23.30 ± 2.61	23.33 ± 2.59	23.18 ± 2.67	0.118
Current year of study:				
Preparatory year	71	51 (2.7)	20 (3.9)	<0.001**
Year 1	262	207 (10.8)	55 (10.8)	
Year 2	258	188 (9.8)	70 (13.7)	
Year 3	345	253 (13.2)	92 (18)	
Year 4	540	460 (24)	80 (15.7)	
Year 5	734	581 (30.3)	153 (29.9)	
Internship	220	179 (9.3)	41 (8)	
Steady financial income				
Yes	854	673 (35.1)	181 (35.4)	0.883
No	1,576	1,246 (64.9)	330 (64.6)	
Living status		1,859 (96.9)	472 (92.4)	<0.001**
With family	2,331		39 (7.6)	
Alone	99	60 (3.1)		
Family member and/or friend has COVID-19				0.016*
Yes	45	29 (1.5)	16 (3.1)	
No	2,385	1,890 (98.5)	495 (96.9)	
Internal displacement				0.063
Yes	294	220 (11.5)	74 (14.5)	
No	2,127	1,692 (88.5)	435 (85.5)	
PHQ-9 score (mean ± SD)	9.76 ± 6.34	9.86 ± 6.31	9.38 ± 6.44	0.913
GAD-7 score (mean ± SD)	7.22 ± 5.08	7.35 ± 5.06	6.75 ± 5.13	0.848

*Significant at *P* < 0.05. **Significant at *P* < 0.001.

### Mental Health Assessments

#### Anxiety

Anxiety symptoms were assessed using the GAD-7 scale, and participants were classified into four grades, as shown in Table 2. The GAD-7 score ranged from 0 to 21, with a median of 6 (IQR, 4–10), while the mean (+ SD) score of anxiety symptoms determined by the GAD-7 was 7.2 (5.1). A total of 268 (11%) students had a GAD-7 score of ≥15, which is indicative of moderate to severe anxiety. However, according to the GAD-7 grades categories, 862 (35.5%) medical students had no signs or symptoms of anxiety. While 1,568 (64.5%) had different degrees of anxiety: mild, 910 (37.5%); moderate, 390 (16%); and severe, 268 (11%). Table 3 demonstrates the relationship between the baseline characteristics and different degrees of anxiety. Anxiety was significantly associated with living status and internal displacement (*P* < 0.05). Students living alone had a higher prevalence of anxiety. In addition, students internally displaced due by the civil war were more statistically associated with anxiety symptoms than were non-displaced students. The gender, age range, year of study, having a steady financial income, and having family members or friends infected with COVID-19 were not statistically associated with anxiety symptoms (*P* > 0.05). Spearman’s correlation was used to assess whether there was a relationship between anxiety score and study characteristics, and there was no statistically significant correlation between the anxiety score determined by the GAD-7 scale and the age (*r*_*s*_ = –0.030, *P* < 0.141). However, there was a statistically significant negative correlation between the anxiety score and the year of study (*r*_*s*_ = –0.41, *P* = 0.042).

**TABLE 2:** Frequencies and percentages of grades of depression and anxiety.

Category	Grade	Frequency	Percentage (%)
Depression (PHQ-9)	Minimal (0–4)	534	22
	Mild (5–9)	855	35.2
	Moderate (10–14)	516	21.2
	Moderately severe (15–19)	289	11.9
	Severe (20–27)	236	9.7
Anxiety (GAD-7)	Normal (0–4)	862	35.5
	Mild (5–9)	910	37.5
	Moderate (10–14)	390	16
	Severe (15–21)	268	11

**TABLE 3:** Univariate analysis of medical students’ anxiety regarding the COVID-19 pandemic.

Variable	Total *n* = 2,430	Anxiety symptoms (GAD-7 ≥ 15)	No anxiety symptoms (GAD-7 < 15)	χ 2	*P*- value
Gender				2.21	0.137
Male	511 (21)	47 (17.5)	464 (21.5)		
Female	1,919 (79)	221 (82.5)	1,698 (78.5)		
Age				1.47	0.224
<24	1,266 (52.1)	149 (55.6)	1,117 (51.7)		
≥24	1,164 (47.9)	119 (44.4)	1,045 (48.3)		
Current year of study:				5.58	0.472
Preparatory year	71 (2.9)	6 (2.2)	65 (3)		
Year 1	262 (10.8)	33 (12.3)	229 (10.6)		
Year 2	258 (10.6)	28 (10.4)	230 (10.6)		
Year 3	345 (14.2)	45 (16.8)	300 (13.9)		
Year 4	540 (22.2)	63 (23.5)	477 (22.1)		
Year 5	734 (30.2)	76 (28.4)	658 (30.4)		
Internship	220 (9.1)	17 (6.3)	203 (9.4)		
Steady financial income				0.187	0.066
Yes	854 (35.1)	91 (34)	763 (35.3)		
No	1,576 (64.9)	177 (66)	1,399 (64.7)		
Living status				3.969	0.046*
With family	2,331(95.9)	251 (93.7)	2,080 (96.2)		
Alone	99 (4.1)	17 (6.3)	82 (3.8)		
Family member and/or acquaintance has COVID-19				0.957	0.328
Yes	45 (1.9)	7 (2.6)	38 (1.8)		
No	2,385 (98.1)	261 (97.4)	2,124 (98.2)		
Internal displacement				12.18	<0.001*
Yes	294 (12.1)	50 (18.7)	244 (11.3)		
No	2,127 (87.9)	218 (81.3)	1,918 (88.7)		

*Significant at *P* < 0.05.

#### Depression

The PHQ-9 scale assessed depression and participants were classified into five grades, as shown in Table 2. The PHQ-9 score ranged from 0 to 21, with a median of 9 (IQR, 5–14), while the mean (+ SD) score of depressive symptoms determined by PHQ-9 was 9.7 (6.3). A total of 525 (21.6%) students had a PHQ-9 score of ≥15, which is indicative of moderate to severe depression. Additionally, 855 (35.2%) of the medical students had mild (PHQ 5–9) depressive symptoms. Suicidal ideation was present in 552 (22.7%) students, as follows: 342 (14.1%), several days; 90 (3.7%), more than half of the days, and 120 (4.9%), nearly every day. Table 2 provides the frequencies of each grade of depression according to the PHQ-9 categories. Depression was only statistically associated with year of study (*P* = 0.009), where medical students in higher years of study had a higher prevalence of depressive symptoms compared to those in earlier years. However, gender, age range, steady financial income, living status, internal displacement, and having a family member or friend infected with COVID-19 were not statistically associated with depressive symptoms (*P* > 0.05). Spearman’s correlation was used to assess whether there was a relationship between the PHQ-9 depressive score and age and year of study. The results showed that there was a statistically significant negative correlation between the year of study and depression score (*r*_*s*_ = –0.76, *P* < 0.001). For the age of the participants, there was a statistically significant negative correlation between age and depression score (*r_*s*_* = –0.054, *P* < 0.008). Table 4 presents the depression grades of study participants in association with COVID-19.

**TABLE 4:** Univariate analysis of medical students’ depression regarding the COVID-19 pandemic.

Variable	Total n = 2,430	Depressive Symptoms (PHQ-9 =15)	No Depressive Symptoms (PHA-9 <15)	χ 2	*p*-value
Gender				2.25	0.134
Male	511 (21)	98 (18.7)	413 (21.7)		
Female	1,919 (79)	427 (81.3)	1,492 (78.3)		
Age				3.32	0.68
<24 =24	1,266 (52.1)	233 (44.4)	931 (48.9)		
	1,164 (47.9)	292 (55.6)	974 (51.1)		
Current year of study:				17.06	0.009*
Preparatory year	71 (2.9)	16 (3)	55 (2.9)		
Year 1	262 (10.8)	59 (11.2)	203 (10.7)		
Year 2	258 (10.6)	52 (9.9)	206 (10.8)		
Year 3	345 (14.2)	100 (19)	245 (12.9)		
Year 4	540 (22.2)	106 (20.2)	434 (12.9)		
Year 5	734 (30.2)	157 (29.9)	577 (30.3)		
Internship	220 (9.1)	35 (6.7)	185 (9.7)		
Steady financial income				0.13	0.717
Yes	854 (35.1)	181 (34.5)	673 (35.3)		
No	1,576 (64.9)	344 (65.5)	1,232 (64.7)		
Living status				1.95	0.16
With family	2,331 (95.9)	498 (94.9)	1,833 (96.2)		
Alone	99 (4.1)	27 (5.1)	72 (3.8)		
Family member and/or acquaintance has COVID-19				2.44	0.118
Yes	45 (1.9)	14 (2.7)	31 (1.6)		
No	2,385 (98.1)	11 (97.3)	1,874 (98.4)		
Internal displacement				1.64	0.2
Yes	294 (12.1)	72 (13.7)	222 (11.7)		
No	2,127 (87.9)	453 (86.3)	1,683 (88.3)		

*Significant at *P* < 0.05.

## Discussion

The COVID-19 pandemic and civil war in Libya has likely had psychological effects on medical students, which can be demonstrated by the high level of anxiety and depression shown in this study. The study determined the psychological status of medical students during the civil war conflicts and the COVID-19 pandemic in Libya. The results demonstrated that 64.5% of students had different degrees of anxiety. Of these, about 11, 16, and 37.5% reported severe, moderate, and mild anxiety symptoms, respectively. In addition, 21.6% had moderate to severe depression symptoms, and suicidal ideation was present in 552 (22.7%) participants.

The high level of anxiety and depression might be related to the civil war (Wells et al., 2011), and overwhelming and exacerbating news that increases their fear of the virus (Ayittey et al., 2020), transmission to family or friends, virus complications, and psychological pressure due to quarantine and isolation (Burtscher et al., 2020; Cao et al., 2020; Molica et al., 2020; Xiao, 2020).

Libyan medical students are exposed to high levels of psychological stress owing to civil war conflicts, resulting in a higher prevalence of anxiety and depression. It is important to note that 294 of the medical students who answered the survey have been internally displaced owing to living in a conflict zone. These students have had to leave their homes and move to a relative’s house or their families have had to find another place to live temporarily. This increases psychological stress; these students’ families will be under higher financial pressure owing to them being forced to rent, and due to the fact that these students are at high risk of being kidnapped or killed and their home and belongings stolen or destroyed in the conflict.

A previous study conducted on Chinese college students during the COVID-19 outbreak demonstrated that 0.9, 2.7, and 21.3% had severe, moderate, and mild anxiety, respectively (Cao et al., 2020); this is lower than our study findings. However, Cao et al. (2020) focused on undergraduate students with no emphasis on the year of study, which varies according to our study findings, as it can play a role in terms of anxiety and depression levels; medical students in higher study years display greater stress and anxiety due to the increased work and study load (Chandavarkar et al., 2007). According to our study, having family members or friends infected with COVID-19 was not significantly associated with anxiety, which is not consistent with previous reports (Cao et al., 2020; Ren et al., 2020). A previous meta-analysis of 69 studies on anxiety among medical students found a pooled prevalence of anxiety of 33.8%, which is similar to our study; the study demonstrated a higher prevalence among Middle Eastern and Asian medical students, which might be due to cultural issues and the stigmatization of mental disorders (Quek et al., 2019).

Our study showed that depression symptoms were associated with the year of the study; those in a higher year of their studies demonstrated a higher prevalence. This is similar to previous studies in Nepal, India, Pakistan, and Thailand, which have also reported that students in higher academic years demonstrated a higher prevalence of psychological morbidities (Supe, 1998; Saipanish, 2003; Shaikh et al., 2004; Sreeramareddy et al., 2007).

Our study showed that 525 (21.6%) of the students were depressed, and suicidal ideation appeared in 552 (22.7%). A previous study in Vietnamese students, which was regarded to have a higher prevalence rate than previous reports, indicated a 15.2% prevalence of depression and 7.7% prevalence of suicidal ideation (Pham et al., 2019). In a recent study among 2,562 Saudi medical students with a similar gender distribution, a total of 15.9% were found to have moderately severe (PHQ-9 = 15–19) depressive symptoms, while 11.6% had severe (PHQ-9 = 20–27) depressive symptoms, which is similar to our results (Alharbi et al., 2018). In a study conducted in the United Arab Emirates, 32.1% of medical students showed evidence of psychiatric distress (Ahmadi et al., 2012). Using the depression, anxiety, and stress scale (DASS-21), another study on Syrian medical students found that the prevalence of depressive symptoms was up to 60.6% and that of anxiety 35.1%, which were higher than those in our study. They also found a higher prevalence among those with “insufficient” personal income (Al Saadi et al., 2017). In Iraq, prevalence of anxiety and depression of 62.5 and 52.1%, respectively, using the DASS-21 questionnaire, were reported (Rasheed and Hussein, 2019). Both Iraq and Syria have similar life stressors to those in Libya, with higher prevalence of anxiety and depression among medical students.

In a systemic review and meta-analysis of 167 cross-sectional studies and 16 longitudinal studies regarding medical students’ depression and suicidal ideation, a prevalence of depression of between 9.3 and 55.9% was revealed across the studies. The pooled prevalence of suicidal ideation was present in 11.1% of the students among the included studies (Rotenstein et al., 2016) which is lower than our study where we found that 22.7% of participants have suicidal ideation.

The strengths of this study are in its adequate sample size and wide representation from 15 different universities and colleges around Libya. Moreover, this study focused on medical students’ anxiety and depression during the COVID-19 pandemic, with special emphasis on several factors, such as internal displacement, financial, and conflict-related factors that are relevant to the students’ current state, with special circumstances regarding the civil war and ongoing conflict.

One of the limitations of this study is its cross-sectional design, which prevents the building of causal relationships. Another limitation is the predominance of the female gender in the study, which may affect the distribution of the results. Furthermore, there might be a selection and interview bias, as the medical students received the survey by email and social media. In addition, this study did not address other factors, such as family history of mental illness, nor other socio-demographic factors, such as emotional trauma.

## Conclusion

Our study highlights that the mental health of medical students in Libya is at risk, especially under the stressful conditions of the civil war and COVID-19 outbreak. In addition to the COVID-19 pandemic, this can be attributed to several factors, including the year of study, age, psychological stress due to the civil war, living with family members or friends with COVID-19, internal displacement due to conflict, and living either with family or alone. These stressors may have long-term effects on their future careers as doctors, which necessitates combined efforts and determined actions to support medical students and provide help for their families. The government should implement strategies aimed at providing mental health support for students to improve healthcare outcomes and decrease the risk of suicide, training attrition, and long-term mental illness during this critical time.

## Data Availability Statement

The datasets used and/or analyzed during the current study are available from the corresponding author on reasonable request.

## Ethics Statement

The study was approved by the Bioethics Committee at the Biotechnology Research Center in Libya.

## Author Contributions

ME analyzed, interpreted the data, and wrote the first draft of the manuscript. Each author took part in the design of the study, contributed to data collections, participated in reviewing the manuscript. All authors read and approved the final manuscript.

## Conflict of Interest

The authors declare that the research was conducted in the absence of any commercial or financial relationships that could be construed as a potential conflict of interest.

## References

[B1] Abuazza A. J. I. P. (2013). The Arab Spring movement: a catalyst for reform at the psychiatric hospital in Tripoli, Libya. *Int. Psychiatry* 10 56–58. 10.1192/s1749367600003842 31507734 PMC6735120

[B2] Ahmadi J. Galal Ahmed M. Ali Bayoumi F. Abdul Moneenum A. Alshawa H. (2012). Mental health of Dubai medical college students. *Iran. J. Psychiatry Behav. Sci.* 6 79–83. 24644486 PMC3940017

[B3] Al Saadi T. Zaher Addeen S. Turk T. Abbas F. Alkhatib M. (2017). Psychological distress among medical students in conflicts: a cross-sectional study from Syria. *BMC Med. Educ.* 17:173. 10.1186/s12909-017-1012-2 28931387 PMC5607487

[B4] Alharbi H. Almalki A. Alabdan F. Haddad B. (2018). Depression among medical students in Saudi medical colleges: a cross-sectional study. *Adv. Med. Educ. Pract.* 9 887–891. 10.2147/amep.s182960 30584384 PMC6287521

[B5] Ayittey F. K. Ayittey M. K. Chiwero N. B. Kamasah J. S. Dzuvor C. (2020). Economic impacts of Wuhan 2019-nCoV on China and the world. *J. Med. Virol.* 92 473–475. 10.1002/jmv.25706 32048740 PMC7166799

[B6] Boland J. W. Dikomitis L. Gadoud A. (2016). Medical students writing on death, dying and palliative care: a qualitative analysis of reflective essays. *BMJ Support. Palliat. Care* 6 486–492. 10.1136/bmjspcare-2016-001110 27486145

[B7] Burtscher J. Burtscher M. Millet G. P. (2020). (Indoor) isolation, stress and physical inactivity: vicious circles accelerated by Covid-19? *Scand. J. Med. Sci. Sports* 30 1544–1545. 10.1111/sms.13706 32374894 PMC7267366

[B8] Cao W. Fang Z. Hou G. Han M. Xu X. Dong J. (2020). The psychological impact of the COVID-19 epidemic on college students in China. *Psychiatry Res.* 287:112934. 10.1016/j.psychres.2020.112934 32229390 PMC7102633

[B9] Chan J. F. Yuan S. Kok K. H. To K. K. Chu H. Yang J. (2020). A familial cluster of pneumonia associated with the 2019 novel coronavirus indicating person-to-person transmission: a study of a family cluster. *Lancet* 395 514–523. 10.1016/s0140-6736(20)30154-9 31986261 PMC7159286

[B10] Chandavarkar U. Azzam A. Mathews C. A. (2007). Anxiety symptoms and perceived performance in medical students. *Depress. Anxiety* 24 103–111. 10.1002/da.20185 16845642

[B11] Chandratre S. (2020). Medical students and COVID-19: challenges and supportive strategies. *J. Med. Educ. Curric. Dev.* 7:2382120520935059. 10.1177/2382120520935059 PMC7315659 32637642

[B12] Charlson F. J. Steel Z. Degenhardt L. Chey T. Silove D. Marnane C. (2012). Predicting the impact of the 2011 conflict in Libya on population mental health: PTSD and depression prevalence and mental health service requirements. *PLoS One* 7:e40593. 10.1371/journal.pone.0040593 22808201 PMC3396632

[B13] Dong E. Du H. Gardner L. (2020). An interactive web-based dashboard to track COVID-19 in real time. *Lancet Infect. Dis.* 20 533–534. 10.1016/s1473-3099(20)30120-1 32087114 PMC7159018

[B14] Elhadi M. Momen A. A. Ali Senussi Abdulhadi O. M. (2020a). A COVID-19 case in Libya acquired in Saudi Arabia. *Travel Med. Infect. Dis.* 10.1016/j.tmaid.2020.101705 [Epub ahead of print]. 32360409 PMC7252057

[B15] Elhadi M. Msherghi A. (2020). COVID-19 and civil war in Libya: the current situation. *Pathog. Glob. Health* 114 230–231. 32432976 10.1080/15575330.2020.1769292 PMC7480619

[B16] Elhadi M. Msherghi A. Alkeelani M. Zorgani A. Zaid A. Alsuyihili A. (2020b). Assessment of healthcare workers’ levels of preparedness and awareness regarding COVID-19 infection in low-resource settings. *Am. J. Trop. Med. Hyg*. 103 828–833. 32563273 10.4269/ajtmh.20-0330 PMC7410457

[B17] Guthrie E. Black D. Bagalkote H. Shaw C. Campbell M. Creed F. (1998). Psychological stress and burnout in medical students: a five-year prospective longitudinal study. *J. R. Soc. Med.* 91 237–243. 10.1177/014107689809100502 9764076 PMC1296698

[B18] Holmes E. A. O’connor R. C. Perry V. H. Tracey I. Wessely S. Arseneault L. (2020). Multidisciplinary research priorities for the COVID-19 pandemic: a call for action for mental health science. *Lancet Psychiatry* 7 547–560. 32304649 10.1016/S2215-0366(20)30168-1 PMC7159850

[B19] Hope V. Henderson M. (2014). Medical student depression, anxiety and distress outside North America: a systematic review. *Med. Educ.* 48 963–979. 10.1111/medu.12512 25200017

[B20] Huang C. Wang Y. Li X. Ren L. Zhao J. Hu Y. (2020). Clinical features of patients infected with 2019 novel coronavirus in Wuhan, China. *Lancet* 395 497–506. 31986264 10.1016/S0140-6736(20)30183-5 PMC7159299

[B21] Johnson S. U. Ulvenes P. G. Øktedalen T. Hoffart A. (2019). Psychometric properties of the general anxiety disorder 7-Item (GAD-7) scale in a heterogeneous psychiatric sample. *Front. Psychol.* 10:1713. 10.3389/fpsyg.2019.01713 31447721 PMC6691128

[B22] Kroenke K. Spitzer R. L. Williams J. B. (2001). The PHQ-9: validity of a brief depression severity measure. *J. Gen. Intern. Med.* 16 606–613. 10.1046/j.1525-1497.2001.016009606.x 11556941 PMC1495268

[B23] Kroenke K. Spitzer R. L. Williams J. B. Monahan P. O. Löwe B. (2007). Anxiety disorders in primary care: prevalence, impairment, comorbidity, and detection. *Ann. Intern. Med.* 146 317–325. 10.7326/0003-4819-146-5-200703060-00004 17339617

[B24] Lai J. Ma S. Wang Y. Cai Z. Hu J. Wei N. (2020). Factors associated with mental health outcomes among health care workers exposed to Coronavirus Disease 2019. *JAMA Netw. Open* 3:e203976. 10.1001/jamanetworkopen.2020.3976 32202646 PMC7090843

[B25] Levis B. Benedetti A. Thombs B. D. (2019). Accuracy of Patient Health Questionnaire-9 (PHQ-9) for screening to detect major depression: individual participant data meta-analysis. *BMJ* 365:l1476. 10.1136/bmj.l1476 30967483 PMC6454318

[B26] Li S. Wang Y. Xue J. Zhao N. Zhu T. (2020). The impact of COVID-19 epidemic declaration on psychological consequences: a study on active Weibo users. *Int. J. Environ Res. Public Health* 17:2032. 10.3390/ijerph17062032 32204411 PMC7143846

[B27] Mahase E. (2020). China coronavirus: WHO declares international emergency as death toll exceeds 200. *BMJ* 368:m408. 10.1136/bmj.m408 32005727

[B28] Miller K. E. Rasmussen A. (2010). War exposure, daily stressors, and mental health in conflict and post-conflict settings: bridging the divide between trauma-focused and psychosocial frameworks. *Soc. Sci. Med.* 70 7–16. 10.1016/j.socscimed.2009.09.029 19854552

[B29] Molica M. Mazzone C. Cordone I. Pasquale A. Niscola P. De Fabritiis P. (2020). SARS-CoV-2 infection anxieties and general population restrictions delay diagnosis and treatment of acute haematological malignancies. *Br. J. Haematol*. 190 e5–e8. 32369605 10.1111/bjh.16785 PMC7267368

[B30] Newnham E. A. Pearson R. M. Stein A. Betancourt T. S. (2015). Youth mental health after civil war: the importance of daily stressors. *Br. J. Psychiatry* 206 116–121. 10.1192/bjp.bp.114.146324 25497299 PMC4312966

[B31] Pham T. Bui L. Nguyen A. Nguyen B. Tran P. Vu P. (2019). The prevalence of depression and associated risk factors among medical students: An untold story in Vietnam. *PLoS One* 14:e0221432. 10.1371/journal.pone.0221432 31430339 PMC6701769

[B32] Quek T. T.-C. Tam W. W.-S. Tran B. X. Zhang M. Zhang Z. Ho C. S.-H. (2019). The global prevalence of anxiety among medical students: a meta-analysis. *Int. J. Environ. Res. Public Health* 16:2735. 10.3390/ijerph16152735 31370266 PMC6696211

[B33] Rasheed A. Hussein A. (2019). Depression, anxiety, and stress among medical students of College of Medicine, Hawler Medical University, Erbil, Iraq. *Zanco J. Med. Sci.* 23 143–152. 10.15218/zjms.2019.019

[B34] Reed R. V. Fazel M. Jones L. Panter-Brick C. Stein A. (2012). Mental health of displaced and refugee children resettled in low-income and middle-income countries: risk and protective factors. *Lancet* 379 250–265. 10.1016/s0140-6736(11)60050-0 21835460

[B35] Reger M. A. Stanley I. H. Joiner T. E. (2020). Suicide mortality and coronavirus disease 2019-A perfect storm? *JAMA Psychiatry.* 10.1001/jamapsychiatry.2020.1060 [Epub ahead of print]. 32275300

[B36] Ren S.-Y. Gao R.-D. Chen Y.-L. (2020). Fear can be more harmful than the severe acute respiratory syndrome coronavirus 2 in controlling the corona virus disease 2019 epidemic. *World J. Clin. Cases* 8 652–657. 10.12998/wjcc.v8.i4.652 32149049 PMC7052559

[B37] Rhouma A. H. Husain N. Gire N. Chaudhry I. B. (2016). Mental health services in Libya. *BJPsych Int.* 13 70–71. 29093908 10.1192/s2056474000001288 PMC5618881

[B38] Rotenstein L. S. Ramos M. A. Torre M. Segal J. B. Peluso M. J. Guille C. (2016). Prevalence of depression, depressive symptoms, and suicidal ideation among medical students: a systematic review and meta-analysis. *JAMA* 316 2214–2236. 10.1001/jama.2016.17324 27923088 PMC5613659

[B39] Ruiz M. A. Zamorano E. Garcia-Campayo J. Pardo A. Freire O. Rejas J. (2011). Validity of the GAD-7 scale as an outcome measure of disability in patients with generalized anxiety disorders in primary care. *J. Affect. Disord.* 128 277–286. 10.1016/j.jad.2010.07.010 20692043

[B40] Saipanish R. (2003). Stress among medical students in a Thai medical school. *Med. Teach.* 25 502–506. 10.1080/0142159031000136716 14522672

[B41] Shaikh B. T. Kahloon A. Kazmi M. Khalid H. Nawaz K. Khan N. (2004). Students, stress and coping strategies: a case of Pakistani medical school. *Educ. Health* 17 346–353. 10.1080/13576280400002585 15848822

[B42] Soled D. Goel S. Barry D. Erfani P. Joseph N. Kochis M. (2020). Medical student mobilization during a crisis: lessons from a COVID-19 medical student response team. *Acad. Med*. 10.1097/ACM.0000000000003401 [Epub ahead of print]. 32282373 PMC7188031

[B43] Spitzer R. L. Kroenke K. Williams J. B. Lowe B. (2006). A brief measure for assessing generalized anxiety disorder: the GAD-7. *Arch. Intern. Med.* 166 1092–1097. 10.1001/archinte.166.10.1092 16717171

[B44] Sreeramareddy C. T. Shankar P. R. Binu V. S. Mukhopadhyay C. Ray B. Menezes R. G. (2007). Psychological morbidity, sources of stress and coping strategies among undergraduate medical students of Nepal. *BMC Med. Educ.* 7:26. 10.1186/1472-6920-7-26 17678553 PMC1951961

[B45] Supe A. N. (1998). A study of stress in medical students at Seth G.S. Medical College. *J. Postgrad. Med.* 44 1–6. 10703558

[B46] Toussaint A. Husing P. Gumz A. Wingenfeld K. Harter M. Schramm E. (2020). Sensitivity to change and minimal clinically important difference of the 7-item Generalized Anxiety Disorder Questionnaire (GAD-7). *J. Affect. Disord.* 265 395–401. 10.1016/j.jad.2020.01.032 32090765

[B47] Ullah R. Amin S. (2020). The psychological impact of COVID-19 on medical students [Letter]. *Psychiatry Res.* 288 113020–113020. 10.1016/j.psychres.2020.113020 32315888 PMC7160641

[B48] UNHCR (2020). *Libya - UNHCR.* Available online at: https://www.unhcr.org/en-us/libya.html (accessed May 1, 2020).

[B49] Urtasun M. Daray F. M. Teti G. L. Coppolillo F. Herlax G. Saba G. (2019). Validation and calibration of the patient health questionnaire (PHQ-9) in Argentina. *BMC Psychiatry* 19:291. 10.1186/s12888-019-2262-9 31533674 PMC6751851

[B50] Wang C. Pan R. Wan X. Tan Y. Xu L. Ho C. S. (2020). Immediate Psychological Responses and Associated Factors during the Initial Stage of the 2019 Coronavirus Disease (COVID-19) Epidemic among the General Population in China. *Int. J. Environ. Res. Public Health* 17:1729. 10.3390/ijerph17051729 32155789 PMC7084952

[B51] Wells T. S. Miller S. C. Adler A. B. Engel C. C. Smith T. C. Fairbank J. A. (2011). Mental health impact of the Iraq and Afghanistan conflicts: a review of US research, service provision, and programmatic responses. *Int. Rev. Psychiatry* 23 144–152. 10.3109/09540261.2011.558833 21521083

[B52] Williams C. M. Wilson C. C. Olsen C. H. (2005). Dying, death, and medical education: student voices. *J. Palliat. Med.* 8 372–381. 10.1089/jpm.2005.8.372 15890048

[B53] Wolf T. M. (1994). Stress, coping and health: enhancing well-being during medical school. *Med. Educ.* 28 8–17. 10.1111/j.1365-2923.1994.tb02679.x 8208174

[B54] Wolf T. M. Faucett J. M. Randall H. M. Balson P. M. (1988). Graduating medical students’ ratings of stresses, pleasures, and coping strategies. *J. Med. Educ.* 63 636–642. 10.1097/00001888-198808000-00008 3398019

[B55] World Health Organization (2020). *Director-General’s remarks at the media briefing on 2019-nCoV on 11 February 2020.* Geneva: WHO.

[B56] Xiao C. (2020). A novel approach of consultation on 2019 Novel Coronavirus (COVID-19)-related psychological and mental problems: structured letter therapy. *Psychiatry Investig.* 17 175–176. 10.30773/pi.2020.0047 32093461 PMC7047000

[B57] Zeiton M. (2011). Frontline medicine: inside Libya. *Lancet* 378 756–757. 10.1016/s0140-6736(11)61360-3 21877328

[B58] Zhou F. Yu T. Du R. Fan G. Liu Y. Liu Z. (2020). Clinical course and risk factors for mortality of adult inpatients with COVID-19 in Wuhan, China: a retrospective cohort study. *Lancet* 395 1054–1062. 10.1016/s0140-6736(20)30566-3 32171076 PMC7270627

[B59] Zhou P. Yang X. L. Wang X. G. Hu B. Zhang L. Zhang W. (2020). A pneumonia outbreak associated with a new coronavirus of probable bat origin. *Nature* 579 270–273. 10.1038/s41586-020-2012-7 32015507 PMC7095418

